# Proton-coupled electron transfer in the electrocatalysis of CO_2_ reduction: prediction of sequential *vs.* concerted pathways using DFT†
†Electronic supplementary information (ESI) available. See DOI: 10.1039/c6sc02984a



**DOI:** 10.1039/c6sc02984a

**Published:** 2016-08-22

**Authors:** Adrien J. Göttle, Marc T. M. Koper

**Affiliations:** a Leiden Institute of Chemistry , Leiden University , PO Box 9502 , 2300 RA Leiden , The Netherlands . Email: m.koper@chem.leidenuniv.nl

## Abstract

Herein we investigate computationally in detail the mechanism of the formation of the carboxylate adduct during the electroreduction of CO_2_ in water catalysed by cobalt porphyrin complexes. Specifically, we address qualitatively the competition between the concerted and sequential pathways for the proton-coupled electron transfer. We use a simple methodology for accurate computation of the p*K*_a_ of the neutral and anionic carboxylate intermediates, [CoP–COOH] and [CoP–COOH]^–^ (where CoP is a cobalt porphine complex), based on the isodesmic proton-exchange reaction scheme. The predicted values are used as in input for a theoretical model that describes the transition between the sequential and concerted pathways. The activation of the sequential pathway (ET–PT) that leads to the formation of the neutral [CoP–COOH] intermediate at pH ≈ 3.5 (p*K*_a_[CoP–COOH] = 3.5 ± 0.4), as predicted by the calculations, is in good agreement with the drastic increase in the faradaic efficiency of the CO_2_ reduction reaction towards CO at pH = 3 compared to pH = 1, as experimentally observed. This confirms the existence of the CO_2_ anionic adduct [CoP–CO_2_]^–^ as a viable intermediate at pH = 3 and its crucial role for the pH dependence of the faradaic efficiency for the CO_2_ reduction. The analysis also shows that when the pH is significantly higher than the p*K*_a_ of the neutral carboxylate adduct, the CO_2_ reduction has to go through an alternative pathway with the formation of the anionic carboxylate intermediate [CoP–COOH]^–^. It is formed through a concerted proton–electron transfer step from the anionic CO_2_ adduct [CoP–CO_2_]^–^ when the pH is below ∼8.6 (p*K*_a_[CoP–COOH]^–^ = 8.6 ± 0.4). At pH ≈ 8.6 and above, another decoupled ET–PT is predicted to take place, leading to the formation of a dianionic CO_2_ adduct [CoP–CO_2_]^2–^.

## Abstract 2

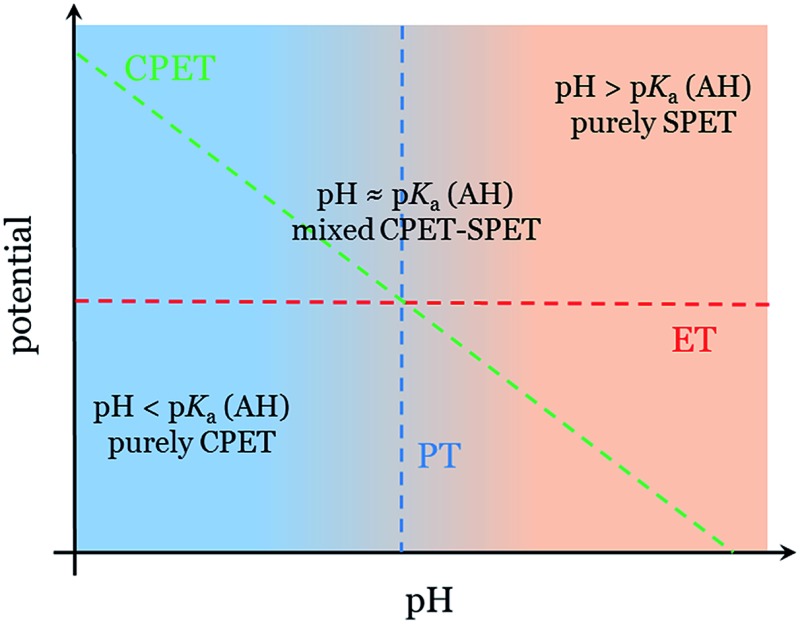
We provide a complete and computationally detailed picture of the mechanism of the initial stages of the electrocatalytic reduction of CO_2_ in water catalysed by cobalt porphyrin complexes.

## 1. Introduction

The reduction of carbon dioxide into valuable products is a research area that currently attracts significant attention within the field of environmental and energy science.^1^ Successful achievements in this highly topical theme could have a drastic impact on modern society, especially by decreasing its dependence on fossil fuels.^2,3^ Still, an efficient system is currently far from realized and progress in this area is closely related to the development of catalysts that are efficient, stable and contain cheap and abundant non-noble metals.^4^

The unravelling of important aspects of the electrochemical CO_2_ reduction reaction (CO_2_RR) mechanism for various type of catalysts using first-principle electronic-structure calculations has provided valuable insights.^5–17^ So far, these investigations have mainly focused on the determination of overpotentials and product selectivity by computation of the thermodynamic energy profiles along the possible reaction pathways. An important mechanistic aspect that is typically not dealt with in these first-principles calculations is the possibility for proton-coupled electron transfers (PCET) to follow pathways where the electron and the proton are either transferred sequentially (sequential proton–electron transfer: SPET) or concertedly (concerted proton–electron transfer: CPET). It is typically believed that the selection between CPET and SPET pathways is closely related to the nature of the catalyst. For molecular catalysts decoupled ET and PT steps are expected, whereas for solid metallic electrocatalysts one assumes CPET steps. However, there is growing evidence for the importance of SPET pathways also on metallic electrocatalysts,^18–25^ and therefore a complete picture must consider both sequential and concerted PCET steps in the whole reaction mechanism. From the computational point of view, the heterogeneous electrocatalysis community typically employs the so-called computational hydrogen electrode (CHE) methodology introduced by Nørskov *et al.*,^26^ which has been very successful in predicting the thermodynamics of CPET steps. However, the CHE methodology cannot account for SPET pathways. Up to now, there is no simple and accurate computational methodology available to systematically address the selection or competition between SPET and CPET pathways in electrocatalytic mechanisms.

In this work, we focus on a concrete example in CO_2_RR catalysis where it has been experimentally supported that the selectivity between CPET and SPET pathways matters: the formation of the carboxylate adduct (*–COOH) and its further reduction (at low pH) and decomposition (at higher pH) to CO during CO_2_RR in water catalysed by a cobalt protoporphyrin IX complex immobilized on an inert pyrolytic graphite electrode.^27^ The neutral *COOH intermediate is assumed to be formed after the addition of the first proton–electron pair and its subsequent reduction leads to the formation of CO or further reduced species (formaldehyde, methanol or methane). We have found that the faradaic efficiency of CO_2_RR on this immobilized molecular catalyst features a drastic change from pH = 1 to pH = 3, with CO production becoming competitive at pH = 3 with the concomitant but undesirable hydrogen evolution reaction (HER). This observation made us postulate the existence of an anionic CO_2_ adduct, capable of subtracting a proton from a nearby water molecule, thereby allowing a high turnover to be maintained for the CO_2_RR in a proton-poorer environment compared to the HER reaction (which is proton diffusion limited at pH = 3). The existence of this intermediate implies a decoupling between ET and PT of the initial PCET.

As mentioned, first-principles studies of the CO_2_RR mechanism on metallic catalysts in water assume that the formation of the neutral carboxylate adduct follows a CPET pathway,* + CO_2_ + H^+^ + e^–^ → *–COOHwhereas studies dealing with molecular catalysts typically assume a SPET (ET–PT) pathway. Such a SPET mechanism was also suggested from a recent theoretical study from our group on CO_2_RR catalysed by cobalt porphyrin complexes:^15^* + CO_2_ + e^–^ → *–CO_2_^–^*–CO_2_^–^ + H^+^ → *–COOHIn our recent DFT study,^15^ we showed with the CHE methodology that the potential limiting step of the CORR to CO is the formation of the neutral carboxylate intermediate (subsequent reduction is predicted to be exothermic). The computed onset potential of –0.43 V (*vs.* a reversible hydrogen electrode) agrees well with the experimental onset potential. It is noteworthy that in both mechanisms (concerted and sequential), in addition to the transfer of the proton–electron pair, there is the association of the CO_2_ with the catalyst. Here, * stands for the active site of the molecular catalyst (metal centre) and e^–^ for the electron of the initially reduced catalyst responsible for the actual reduction of CO_2_. This notation follows more closely the notation used for PCET steps in heterogeneous electrocatalysis but one has to keep in mind that from the molecular electrocatalysis point-of-view, the electron actually “flows” to the substrate in two steps with the initial reduction of the catalyst, followed by subsequent ligation by the adduct with a corresponding charge redistribution. After the formation of the CO_2_ anionic adduct, different scenarios are possible depending on the catalyst and on the experimental conditions (applied potential, pH). In particular, from the work of Leung *et al.*, a PCET pathway is predicted to take place at neutral pH from the CO_2_ anionic adduct to form an anionic carboxylate intermediate, which can readily decompose to form CO.^6^

Our specific aim in this work is to introduce a simple and general methodology to elucidate the mechanism of the formation of the carboxylate adduct for a simple porphyrin catalyst (cobalt porphine complex: CoP) taking into account the active role of the pH in the selectivity between the CPET and SPET pathways, and also in the selectivity between the possible charged states of the carboxylate adduct (neutral or anionic). The key ingredient of this methodology is the calculation of the acid–base equilibrium constant, which is a necessary quantity to compare the thermodynamics of the CPET and SPET pathways and to rationalize the transition between sequential and concerted PCET. To this end, we base ourselves on the accurate computation of p*K*_a_ values of the neutral [CoP–COOH] and anionic [CoP–COOH]^–^ species using a simple method known as the isodesmic proton exchange reaction scheme (IPER).^28^ We predict that at pH ≈ 3.5 and higher, the formation of the neutral [CoP–COOH] intermediate follows preferentially the sequential pathway with the formation of the anionic CO_2_ adduct [CoP–CO_2_]^–^ as a viable intermediate. At much higher pH, another pathway takes place that results in the formation of the anionic [CoP–COOH]^–^ intermediate, followed by another SPET at pH ≈ 8.3 and higher. Beyond the specific example investigated in this work, the simple methodology introduced in this paper can be applied to any molecular or metallic electrocatalyst for the systematic prediction of the selectivity between SPET and CPET, and thereby allows the calculation of reaction schemes beyond the CHE methodology.

## 2. Theory and methodology

### 2.1 Theoretical background

**Fig. 1:**
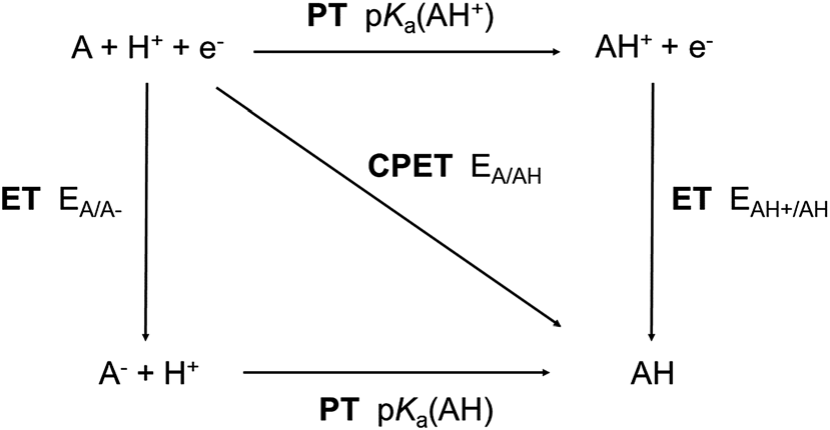
Possible pathways for a proton-coupled electron transfer (ET: electron transfer, PT: proton transfer) and the thermodynamic quantities associated with the reaction steps.

PCET reactions are ubiquitous in chemistry.^29^ The well-known square scheme depicted in Fig. 1 illustrates the possible scenarios for PCET reactions in general: the CPET pathway corresponding to the “diagonal” path, and SPET pathways corresponding to “off-diagonal” paths. Significant attention has been devoted to the fundamental description and the experimental implications of these pathways.^29–34^

In order to address the selectivity between these pathways, one has to take into account their relative kinetics and thermodynamics. A recently proposed theoretical model describes the transition between SPET and CPET pathways by providing analytical expressions for the activation energies of the ET, PT and CPET steps that include as parameters both thermodynamic quantities and reorganization energies.^35^ Depending on the relative values of these parameters, one can distinguish when a certain pathway, CPET or SPET, is preferred over the other due to a lower activation barrier(s). Assuming outer-sphere charge transfer, the rate constants of the ET, PT and CPET steps are given by the following Marcus-type expressions:1
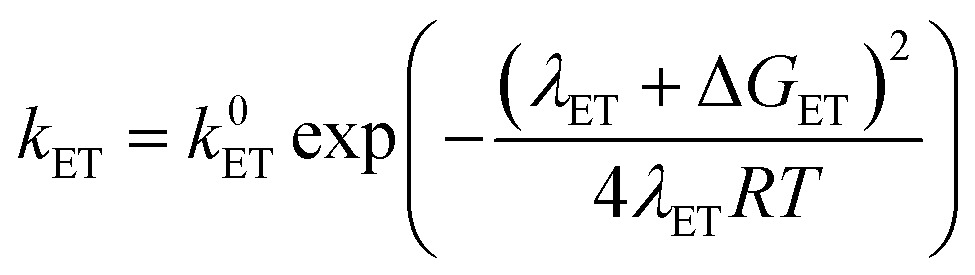

2
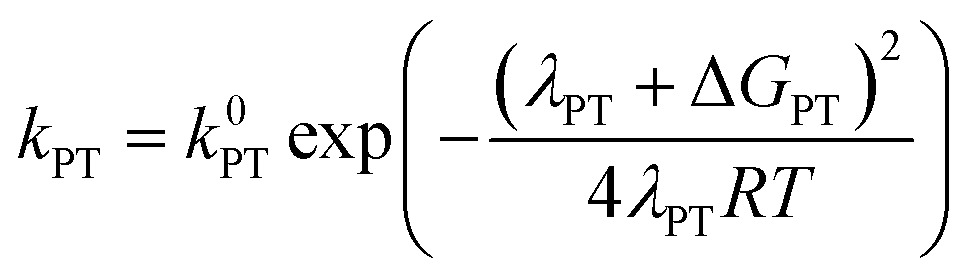

3
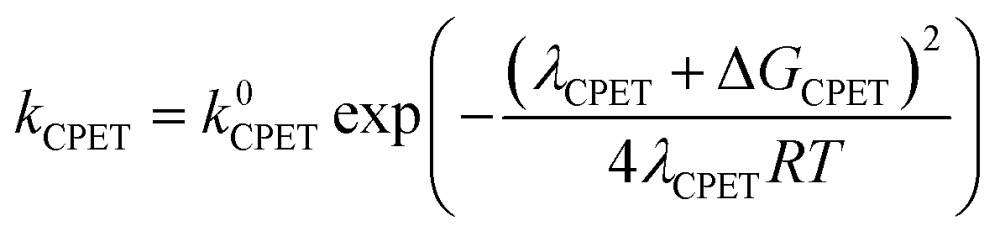
In these equations, the *k* ^0^ parameters are pre-exponential factors, the *λ* variables are the reorganization energies and the Δ*G* values are the free reaction energies of the separate ET, PT and CPET reaction steps. The above expressions for outer-sphere charge transfer may not be directly applicable to PCET reactions involving catalysts, but they are useful in that they clearly distinguish between the impact of activation-related parameters (*λ* values) and thermodynamics-related parameters (Δ*G* values). Explicit expressions for the solvent-related part of the reorganization energies *λ*_ET_ and *λ*_PT_ based on a continuum representation of the solvent may be found in the literature.^36–39^ The expression for *λ*_CPET_ is given by:^40^

4 *λ*_CPET_ = *λ*_ET_ + *λ*_PT_ + 2*λ̄*

where *λ̄* is the so-called cross reorganization energy, or solvent overlap. This parameter describes the extent to which the solvent reorganization for ET and PT involves the reorganization of the same or different modes. In case there is no overlap between ET and PT modes, *λ̄* = 0. Unfortunately, simple models for *λ̄* do not exist but computational examples have been considered by Hammes-Schiffer *et al*.^41,42^ However, note that if *λ̄* ≈ 0, and if all steps are close to thermodynamic equilibrium, *i.e.* all Δ*G* ≈ 0, the activation energy for CPET is always higher than the activation energies for ET and PT, and hence under those circumstances SPET is more likely than CPET (Fig. S1† illustrates this particular case).

**Fig. 2:**
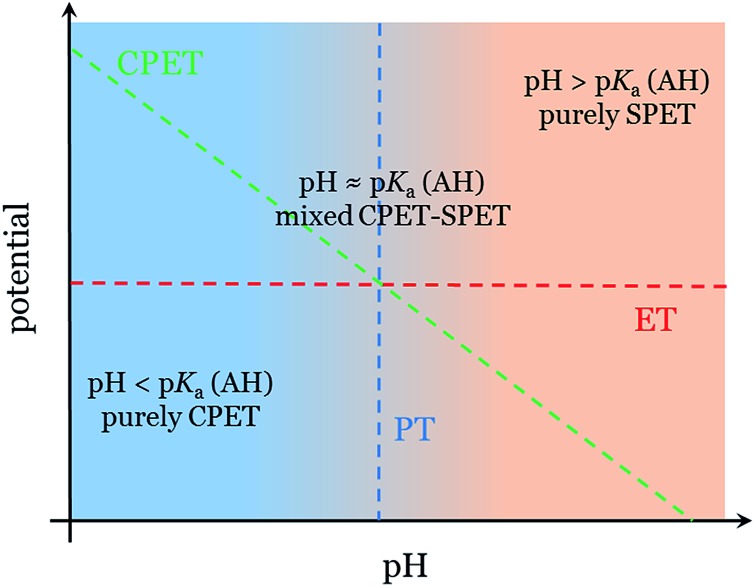
Pourbaix diagram showing the thermodynamic equilibria of the CPET (green, A + H^+^ + e^–^ → AH), ET (red, A + e^–^ → A^–^) and PT (blue, A^–^ + H^+^ → AH) steps.

The thermodynamics of the three reaction steps ET, PT and CPET scale differently with pH and, thus, the pH also impacts differently the kinetics of these steps due to the Δ*G* term in eqn (1)–(3). This is illustrated in Fig. 2, which represents the thermodynamic equilibria of the various steps in a Pourbaix diagram. When pH = p*K*_a_(AH), all steps are equilibrated and the competition between CPET and SPET pathways is governed by the reorganization energies (see above). The thermodynamics of the CPET and PT steps are sensitive to the pH and so are their kinetics. More precisely, their rate increases (decreases) when the pH decreases (increases). By contrast, the thermodynamics and kinetics of the ET step are not sensitive to pH. As a result, the pH can modify the competition between the CPET and SPET pathways if eqn (1)–(3) apply (for a reduction reaction). The pH dependence of the competition between the CPET and the decoupled ET–PT pathways, which is relevant for the concrete example studied in this work, will now be discussed in more detail.

To address this pH dependence more quantitatively, it is important to pay attention to the numerical values of the reorganization energies (*λ*_ET_ and *λ*_CPET_) (*λ*_PT_ for the decoupled PT–ET pathways). We find that the position of the pH domains with qualitatively different competition, namely the two pH domains where either the CPET or the SPET takes place exclusively, and the transition region where both pathways compete at comparable rates, is very sensitive to the values of the reorganization energies. When *λ*_CPET_ ≈ *λ*_ET_, the transition region is always centred around pH ≈ p*K*_a_(AH) and spans approximately the pH range p*K*_a_ = –2 to p*K*_a_ = +2 (see Fig. S2a in the ESI†). As a result, in this case, the pH dependence of the CPET–SPET competition can be qualitatively addressed based on the sole knowledge of the p*K*_a_ of the AH species formed following a PCET. This is depicted in Fig. 2 where we illustrate the three relevant pH domains. The transition region is defined by pH ≈ p*K*_a_(AH) for the sake of simplicity but one has to keep in mind that it spans 1–2 pH units around the p*K*_a_(AH) (see above). When the difference between *λ*_CPET_ and *λ*_ET_ increases, specifically when *λ*_CPET_ > *λ*_ET_ as predicted by eqn (4), the position of the transition region can be substantially shifted to lower pH compared to the case where *λ*_CPET_ ≈ *λ*_ET_ (see Fig. S2b in the ESI†). As a result, in general, evaluating the pH dependence of the competition between the CPET and SPET pathways requires the computation of reorganization energies. To fulfil the condition *λ*_CPET_ ≈ *λ*_ET_, strong overlap is required between the ET and PT reaction coordinates (the solvent overlap *λ̄* should have a substantially negative value) and it has been argued that this is typically the case when the directionality for PT and ET is similar.^37,41,42^ Interestingly, strong solvent overlap could in principle lead to a situation for which *λ*_CPET_ < *λ*_ET_ and consequently CPET would be favoured over SPET even if on purely thermodynamic grounds one would expect that SPET is to be the most likely pathway.

### 2.2 Isodesmic proton-exchange reaction scheme

With the isodesmic proton-exchange reaction scheme (IPER), the p*K*_a_ of an acid AH is calculated with respect to the known p*K*_a_ of a reference acid “refH”.^28^

5 AH_sol_^*m*^ + ref_sol_^*n*–1^ → A_sol_^*m*–1^ + refH_sol_^*n*^;  Δ*G*_sol_

6
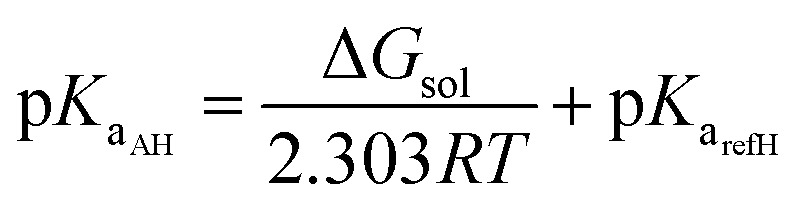
The main purpose of this method is to improve the accuracy of the predictions using continuum solvation models (CSM) by taking advantage of error cancelation, especially regarding solvation energies of ionic species, which has been shown to be the main source of errors with CSM (∼5 kcal mol^–1^).^43–47^ The key point for achieving good predictions is the choice of the reference acid. In practice, references with similar charge distribution and chemical environment in the vicinity of the acid group are considered to perform well and the total charges *m* and *n* in eqn (5) can actually be different without loss of accuracy (the solute–continuum electrostatic interaction is computed based on the local charge distribution).^28,48^ The remainder of the molecule has little impact on the quality of the prediction. Note that attention must be paid to the microsolvation effect on the acid group, in our case the addition of two water molecules was necessary to produce an accurate p*K*_a_ prediction for the anionic carboxylate intermediate, as we will show below. The specificity of the IPER method is that the reaction energy in solution, Δ*G*_sol_, is obtained directly from the computed free energies of the individual species in solution. In other words, all calculations (geometry optimization and finite temperature corrections for these geometries) have been performed with the continuum solvation model. It thus differs from the thermodynamic cycles which requires the computation of gas phase free energies and the solvation free energies. In our case, the calculation of p*K*_a_[CoP–COOH] using thermodynamics cycles methods is compromised by the fact that the neutral CO_2_ adduct is not stable in the gas phase.^15^ Recent studies performed on extensive sets of acids have shown that the IPER method provides p*K*_a_ values with accuracies comparable to the one obtained with thermodynamic cycles and can even outperform them in some cases.^28^

In general, good linear correlations between experimental and computed p*K*_a_ values with proton exchange methods have been observed^28^ for various families of acids and thus, reliable predictions can be derived from them. It is worth noting that the quality of the correlation depends little on the calculation level used for the electronic energies as there is no significant difference between density functional and high-level *ab initio* calculations. Instead, the accuracy of the p*K*_a_ calculation primarily depends on the performance of the implicit solvation model used. However, the parameters of the linear fit obtained are usually far from the ideal behaviour (slope = unity and offset = 0) and, as a consequence, the absolute errors increase with the p*K*_a_ difference with respect to the reference. Small regular absolute errors can be obtained over the p*K*_a_ range investigated by applying a correction that shifts all the computed values so that a new linear fit based on the corrected values matches the ideal behaviour (the mathematical details for the case treated in this study are provided in Fig. S3†).

### 2.3 Computational details

All density functional theory electronic structure calculations were performed with the Gaussian 09 package.^49^ The hybrid functional Perdew, Burke and Ernzerhof (PBE0) was used.^50^ A triple-*ζ* quality basis set that includes one polarization function and augmented with one diffuse function (6-311+G*)^51–53^ was used for non-metallic atoms, and the Stuttgart–Dresden effective core potential MDF10^ ^^54^ together with its corresponding basis set^55^ have been used for the cobalt. The solvent effects are modelled by the universal continuum solvent model based on density commonly called SMD.^43^

## 3. Results and discussion

**Fig. 3:**
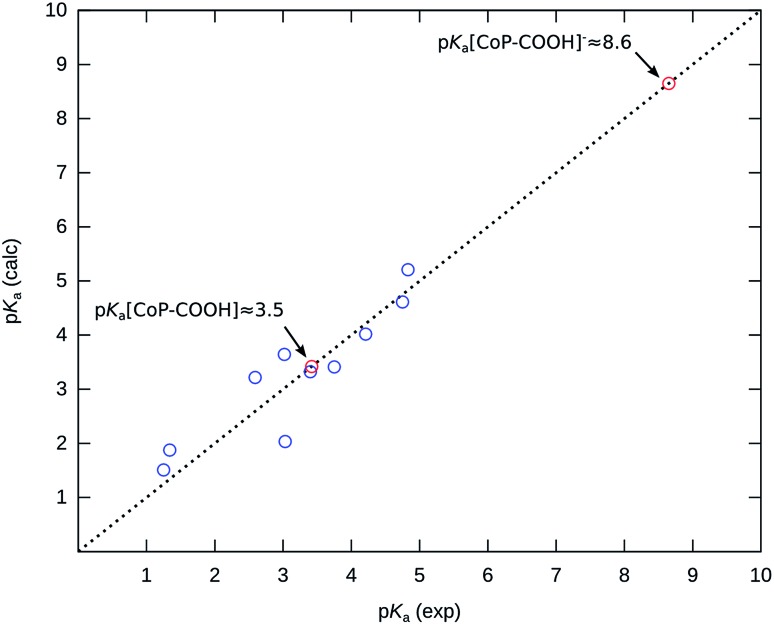
Plot of p*K*_a_ predicted against experimental values for the benchmark series of acids and p*K*_a_ of the carboxylic intermediates [CoP–COOH] and [CoP–COOH]^–^.

Since we want to compute the p*K*_a_ of carboxylic acids ([CoP–COOH] and [CoP–COOH]^–^), we have considered a series of acids of the same family in order to derive the parameters of the linear fit that corresponds to the calculation level used. We have chosen a set of acids with p*K*_a_ spanning the range ∼0.5 (trifluoroacetic acid) to ∼4.8 (butyric acid) and formic acid was arbitrarily chosen as the reference species. A very good linear correlation is found (*r* = 0.983), which demonstrates that formic acid, despite being the simplest carboxylic acid, is a reliable reference. As expected, the parameters are far from their ideal values (slope = 2.03 and offset = –3.52, see Fig. S3a†). After correction, the mean absolute error obtained is ∼0.4 p*K*_a_ unit (Fig. S3b†) which is consistent with the values that have been typically obtained in previous works with other functionals.^28^ One can expect the same accuracy for the p*K*_a_ values predicted for [CoP–COOH] and [CoP–COOH]^–^. The p*K*_a_ values obtained for the series of acids considered together with the values obtained for [CoP–COOH] and [CoP–COOH]^–^ are displayed Fig. 3.

We predict that p*K*_a_[CoP–COOH] ≈ 3.5 ± 0.4 and p*K*_a_[CoP–COOH]^–^ ≈ 8.6 ± 0.4. It is worth noting that the p*K*_a_ value of the anionic [CoP–COOH]^–^ intermediate is significantly far from the values usually observed for carboxylic acids (explained in detail later). The lack of experimental p*K*_a_ values above ∼5 makes the estimation of the accuracy derived from the chosen series of acids (0.4 p*K*_a_ units) less reliable for the extrapolated p*K*_a_[CoP–COOH]^–^. Still, the p*K*_a_ values obtained are in very good agreement with values that have been previously obtained by Leung *et al.* with *ab initio* molecular dynamics (AIMD) simulations (3.8 and 9.0 respectively for [CoP–COOH] and [CoP–COOH]^–^).^6^ Attention must be paid to the structures considered for both intermediates. In particular for the anionic species, the addition of explicit water molecules was necessary to predict a p*K*_a_ value in agreement with the AIMD value. For both [CoP–COOH] and [CoP–COOH]^–^, two isomers are possible depending on the position of the hydrogen of the carboxylic group (H can point towards or away from the porphyrin ring, see Fig. S4†). For the neutral [CoP–COOH], they are almost degenerate and have very similar p*K*_a_ values (3.42 and 3.60). We consider the average of these two values as the p*K*_a_ of this intermediate (p*K*_a_ ≈ 3.5). For the anionic intermediate these two isomers are also relatively close in energy with p*K*_a_ = 5.02 and 5.79, but they are far from the value predicted from AIMD (p*K*_a_ = 9.0). The reason for such a difference is related to a drastic difference in the charge distribution within the complex with and without explicit solvation. According to a natural charge analysis (Table S1†), the charge borne by the carboxylic group for the isomer with the hydrogen pointing towards the porphyrin ring is negligible without any explicit solvent molecules whereas it is ∼–0.5 with the addition of two explicit water molecules (Fig. S5†). This large negative charge on the carboxylic group explains its much higher basicity. The inclusion of microsolvation for the [CoP–COOH]^–^ intermediate changes the shape of the cavity around the acid/base group, and thus the local electrostatic interaction with the continuum is not computed in the same way as the reference which may result in poorer error cancelation or even unexpected artefacts. To address that point, we have computed the p*K*_a_ of the neutral [CoP–COOH] again including microsolvation and compared the value with the one without microsolvation. The difference is negligible (p*K*_a_ values are both ≈3.5), so we can expect that the value obtained for [CoP–COOH]^–^ is reasonable.

**Fig. 4:**
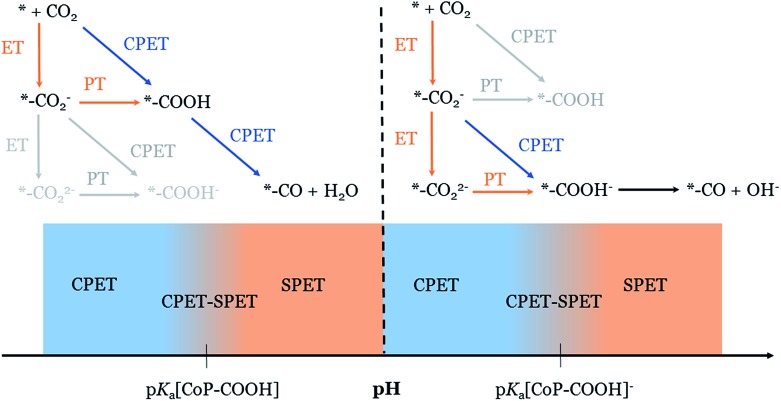
Schematic depiction of the dominant mechanism of the formation of the carboxylate intermediate depending on the pH.

The formation of the neutral carboxylate adduct implies a reorganization of the reactant configuration since it requires the association of the catalyst with CO_2_. This may introduce an additional energetic contribution that may significantly impact the thermodynamics and kinetics of the ET and CPET steps compared to a simple PCET without such adduct formation. However, as far as the cobalt porphine complex used in this work is concerned, we have shown in a previous theoretical DFT study that the association with the reduced catalyst [CoP]^–^ is almost barrierless and thermoneutral.^15^ Therefore, this contribution can reasonably be neglected in our case and the pH is the determinant factor that governs the competition between CPET and SPET (*cf.* 2.1). Still, one should in general pay attention to this effect for other catalysts. The p*K*_a_ value computed for the neutral [CoP–COOH] intermediate allows the pH domains to be determined for which the SPET and/or CPET pathways take place (Fig. 4). In the rest of the discussion, we will use the same definition as in Fig. 2 to define these pH domains (pH < p*K*_a_, pH ≈ p*K*_a_ and pH > p*K*_a_). At a pH below 3.5, the formation of [CoP–COOH] is expected to follow exclusively the CPET pathway. When CO_2_RR is performed at a pH close to 3.5, the SPET and CPET pathways are predicted to co-exist. At this stage of the discussion, it is interesting to use these theoretical predictions to address which mechanism is likely to take place for the pH range investigated in the experimental study.^27^ At pH = 1, the formation of [CoP–COOH] is predicted to follow a purely CPET mechanism (pH < p*K*_a_[CoP–COOH]) whereas at pH = 3 it follows a mixed SPET–CPET mechanism. If we compare the pH dependence predicted for the mechanism of the formation of the neutral [CoP–COOH] and for the experimentally observed faradaic efficiencies of the COR and HER reactions, the similarity is striking. Therefore, this theoretical study supports the assumption that the significant formation of the anionic [CoP–CO_2_]^–^ adduct is a key ingredient for understanding the drastic change in faradaic efficiency of the CO_2_RR to CO, compared to the HER reaction, when the pH changes from 1 to 3. It is also interesting to point out that, using the value of the onset potential value computed with the CHE methodology for the concerted pathway (*i.e.*, –0.43 V *vs.* reversible hydrogen electrode),^15^ the equilibrium potential extrapolated at pH = 3.5, ∼0.63 (–0.059 mV shift per pH unit) is very close to the average experimental redox potential of the catalyst –0.67 (between –0.5 to –0.84 depending on substituent and solvent).^56^

It has been experimentally demonstrated that CO_2_RR on Co-porphyrins can proceed at pH = 7.^57,58^ Under such a condition, the formation of the neutral [CoP–COOH] intermediate is unlikely (p*K*_a_[CoP–COOH] ≪ 7) and an alternative mechanism must take place. Taking into account the large p*K*_a_ value predicted for the anionic [CoP–COOH]^–^ (p*K*_a_ = 8.6), a logical alternative is a pathway with the formation of this intermediate. This latter species is formed after a PCET from the anionic [CoP–CO_2_]^–^ adduct and, like the formation of [CoP–COOH] at lower pH, it can follow either the SPET or CPET pathways. One can predict that [CoP–CO_2_]^2–^ will only be formed when the pH is close to or above p*K*_a_[CoP–COOH] = 8.6, *i.e.* the region where the SPET pathway is predicted to be active (Fig. 4). It results in the following ET–PT sequence for pH > p*K*_a_[CoP–COOH]^–^ ≈ 8.6:*–CO_2_^–^ + e^–^ → *–CO_2_^2–^*–CO_2_^2–^ + H_2_O → *–COOH^–^ + OH^–^For the pH range p*K*_a_[CoP–COOH] < pH < p*K*_a_[CoP–COOH]^–^, the CPET mechanism is predicted to be the most efficient pathway, circumventing the formation of the dianionic [CoP–CO_2_]^2–^ adduct:*–CO_2_^–^ + H_2_O + e^–^ → *–COOH^–^ + OH^–^.

## 4. Conclusion

In this work, we have provided a complete picture of the mechanism of the formation of the carboxylate adduct on a model molecular cobalt porphyrin catalyst that accounts for the possible coupling or decoupling of proton and electron transfer in the initial stages of the electrocatalytic reduction of CO_2_. The mechanism of the formation of the neutral carboxylate adduct [CoP–COOH] is predicted to change from purely CPET to mixed CPET–SPET in the vicinity of pH ≈ 3.5. This value corresponds to the p*K*_a_ of this intermediate. The prediction that the SPET pathway becomes competitive at pH ≈ 3.5 is in agreement with the drastic increase of the CO_2_RR faradaic efficiency towards CO, as experimentally observed at pH = 3, which supports the crucial role of the anionic [CoP–CO_2_]^–^ adduct. At higher pH (a few pH units above pH = 3.5), the formation of the neutral carboxylate adduct [CoP–COOH] is unlikely and the CO_2_RR reaction mechanism is more likely to go through an anionic carboxylate adduct [CoP–COOH]^–^. The formation of this latter species is predicted to take place through a CPET pathway from the anionic CO_2_ adduct [CoP–CO_2_]^–^ when the pH is below 8.6, which corresponds to the p*K*_a_ of [CoP–COOH]^–^. At pH ≈ 8.6 and above, the SPET pathway from [CoP–CO_2_]^–^ is predicted to be active which implies the formation of a dianionic CO_2_ adduct [CoP–CO_2_]^2–^.

Besides the valuable insights into the formation of the carboxylate adduct during CO_2_ reduction as provided in this work, our suggested methodology is also an important step forward in first-principles mechanistic studies of electrocatalytic reactions since it takes into account the important role of the pH in the selection between the CPET and SPET pathways. The simple methodology used in this work (*i.e.* calculation of p*K*_a_ using the IPER method and employment of this value within the framework of the theoretical model that describes the transition between SPET and CPET for PCET reactions)^35^ has been demonstrated to be relevant for systematically addressing the competition between the SPET and CPET pathways, and provides a valuable extension to the computational hydrogen electrode method. In addition to its application to molecular catalysts, it should also be applicable to metallic and other solid-state electrocatalysts, for which there is growing evidence for SPET pathways in important reactions such as CO_2_ and CO reduction, O_2_ reduction, water oxidation, and ammonia oxidation.^37^ However, the computation of the p*K*_a_ of surface adsorbates is likely to be more challenging than for molecular systems as the water is more structured in the vicinity of surfaces (explicit microsolvation is probably necessary) and also the choice of the reference is not straightforward as typically no experimental p*K*_a_ values are available for surface adsorbates.

## References

[cit1] Aresta M., Dibenedetto A., Angelini A. (2014). Chem. Rev..

[cit2] International Energy Agency, Key World Energy Stat., 2015, vol. 2015.

[cit3] McGlade C., Ekins P. (2015). Nature.

[cit4] Lim R. J., Xie M., Sk M. A., Lee J.-M., Fisher A., Wang X., Lim K. H. (2014). Catal. Today.

[cit5] Nielsen I. M. B., Leung K. (2010). J. Phys. Chem. A.

[cit6] Leung K., Nielsen I. M. B., Sai N., Medforth C., Shelnutt J. A. (2010). J. Phys. Chem. A.

[cit7] Tripkovic V., Vanin M., Karamad M., Bjorketun M. E., Jacobsen K. W., Thygesen K. S., Rossmeisl J. (2013). J. Phys. Chem. C.

[cit8] Liu C., Cundari T. R., Wilson A. K. (2012). J. Phys. Chem. C.

[cit9] Cheng D., Negreiros F. R., Apra E., Fortunelli A. (2013). ChemSusChem.

[cit10] Cheng M.-J., Kwon Y., Head-Gordon M., Bell A. T. (2015). J. Phys. Chem. C.

[cit11] Zhang Y.-J., Sethuraman V., Michalsky R., Peterson A. A. (2014). ACS Catal..

[cit12] Song J., Klein E. L., Neese F., Ye S. (2014). Inorg. Chem..

[cit13] Karamad M., Hansen H. A., Rossmeisl J., Norskov J. K. (2015). ACS Catal..

[cit14] Montoya J. H., Shi C., Chan K., Norskov J. K. (2015). J. Phys. Chem. Lett..

[cit15] Shen J., Kolb M. J., Gottle A. J., Koper M. T. M. (2016). J. Phys. Chem. C.

[cit16] Peterson A. A., Abild-Pedersen F., Studt F., Rossmeisl J., Norskov J. K. (2010). Energy Environ. Sci..

[cit17] Peterson A. A., Norskov J. K. (2012). J. Phys. Chem. Lett..

[cit18] Lai S. C. S., Kleijn S. E. F., Ozturk F. T. Z., van R. Vellinga V. C., Koning J., Rodriguez P., Koper M. T. M. (2010). Catal. Today.

[cit19] Kwon Y., Lai S. C. S., Rodriguez P., Koper M. T. M. (2011). J. Am. Chem. Soc..

[cit20] Rodriguez P., Kwon Y., Koper M. T. M. (2011). Nat. Chem..

[cit21] Joo J., Uchida T., Cuesta A., Koper M. T. M., Osawa M. (2013). J. Am. Chem. Soc..

[cit22] Hori Y., Takahashi I., Koga O., Hoshi N. (2002). J. Phys. Chem. B.

[cit23] Schouten K. J. P., Qin Z., Gallent E. P., Koper M. T. M. (2012). J. Am. Chem. Soc..

[cit24] Kortlever R., Shen J., Schouten K. J. P., Calle-Vallejo F., Koper M. T. M. (2015). J. Phys. Chem. Lett..

[cit25] Katsounaros I., Chen T., Gewirth A. A., Markovic N. M., Koper M. T. M. (2016). J. Phys. Chem. Lett..

[cit26] Nørskov J. K., Rossmeisl J., Logadottir A., Lindqvist L., Kitchin J. R., Bligaard T., Jónsson H. (2004). J. Phys. Chem. B.

[cit27] Shen J., Kortlever R., Kas R., Birdja Y. Y., Diaz-Morales O., Youngkook K., Ledezma-Yanez I., Schouten K. J. P., Mul G., Koper M. T. M. (2015). Nat. Commun..

[cit28] Casasnovas R., Ortega-Castro J., Frau J., Donoso J., Munoz F. (2014). Int. J. Quantum Chem..

[cit29] Huynh M. H. V., Meyer T. J. (2007). Chem. Rev..

[cit30] Hammes-Schiffer S., Stuchebrukhov A. A. (2010). Chem. Rev..

[cit31] Solis B. H., Hammes-Schiffer S. (2014). Inorg. Chem..

[cit32] Costentin C., Robert M., Saveant J.-M. (2010). Acc. Chem. Res..

[cit33] Costentin C., Hajj V., Louault C., Robert M., Saveant J.-M. (2011). J. Am. Chem. Soc..

[cit34] Costentin C. (2008). Chem. Rev..

[cit35] Koper M. T. M. (2013). Phys. Chem. Chem. Phys..

[cit36] Costentin C., Robert M., Savéant J.-M. (2006). J. Electroanal. Chem..

[cit37] Koper M. T. M. (2013). Chem. Sci..

[cit38] Marcus R. (1960). Discuss. Faraday Soc..

[cit39] Marcus R. (1965). J. Chem. Phys..

[cit40] Soudackov A., Hammes-Schiffer S. (1999). J. Chem. Phys..

[cit41] Auer B., Soudackov A. V., Hammes-Schiffer S. (2012). J. Phys. Chem. B.

[cit42] Soudackov A. V., Hazra A., Hammes-Schiffer S. (2011). J. Chem. Phys..

[cit43] Marenich A. V., Cramer C. J., Truhlar D. G. (2009). J. Phys. Chem. B.

[cit44] Takano Y., Houk K. N. (2005). J. Chem. Theory Comput..

[cit45] Kelly C. P., Cramer C. J., Truhlar D. G. (2005). J. Chem. Theory Comput..

[cit46] Marenich A. V., Olson R. M., Kelly C. P., Cramer C. J., Truhlar D. G. (2007). J. Chem. Theory Comput..

[cit47] Klamt A., Mennucci B., Tomasi J., Barone V., Curutchet C., Orozco M., Luque F. J. (2009). Acc. Chem. Res..

[cit48] Sastre S., Casasnovas R., Munoz F., Frau J. (2016). Phys. Chem. Chem. Phys..

[cit49] Firchk M. J., Trucks G. W., Schlegel H. B., Scuseria G. E., Robb M. A., Cheeseman J. R., Scalmani G., Barone V., Mennucci B., Petersson G. A., Nakatsuji H., Caricato M., Li H., Hratchian H. P., Izmaylov A. F., Bloino J., Zheng G., Sonnenberg J. L., Hada M., Ehara M., Toyota K., Fukuda R., Hasegawa J., Ishida M., Takajima T., Honda Y., Kitao O., Nakai H., Vreven T., Montgomery J. A., Peralta J. E., Ogliaro F., Bearpark M., Heyd J. J., Brothers E., Kudin K. N., Staroverov V. N., Kobayashi R., Normand J., Raghavachari K., Rendell A., Burant J. C., Iyengar S. S., Tomasi J., Cossi M., Rega N., Millam J. M., Klene M., Knox J. E., Cross J. B., Bakken V., Adamo C., Jaramillo J., Gomperts R., Stratmann R. E., Yzyev O., Austin A. J., Cammi R., Pomelli C., Ochterski J. W., Martin R. L., Morokuma K., Zakrzewky V. G., Voth G. A., Salvador P., Dannenberg J. J., Dapprich S., Daniels A. D., Farkas Ö., Foresman J. B., Ortiz J. V., Cioslowski J. and Fox D. J., Gaussian 09 Revision D01, Gaussian Inc., Wallingford CT, 2009.

[cit50] Adamo C., Barone V. (1999). J. Chem. Phys..

[cit51] Krishnan R., Binkley J., Seeger R., Pople J. (1980). J. Chem. Phys..

[cit52] Frisch M., Pople J., Binkley J. (1984). J. Chem. Phys..

[cit53] Clark T., Chandrasekhar J., Spitznagel G., Schleyer P. (1983). J. Comput. Chem..

[cit54] Dolg M., Wedig U., Stoll H., Preuss H. (1987). J. Chem. Phys..

[cit55] Martin J. M. L., Sundermann A. (2001). J. Chem. Phys..

[cit56] Kadish K., Royal G., Van Caemelbecke E. and Gueletti L., The Porphyrin Handbook, 2000, vol. 9.

[cit57] Furuya N., Matsui K. (1989). J. Electroanal. Chem..

[cit58] Shibata M., Furuya N. (2003). Electrochim. Acta.

