# Supplementary material for: Proton-coupled electron transfer in the electrocatalysis of CO2 reduction: prediction of sequential vs. concerted pathways using DFT
Source: Chem Sci. 2016 Aug 22;8(1):458–65. doi: 10.1039/c6sc02984a (PMC5298188; doi:10.1039/c6sc02984a)
Supplement: Supplementary file 1 [file SC-008-C6SC02984A-s001.pdf]

Electronic Supplementary Material (ESI) for Chemical Science.
This journal is © The Royal Society of Chemistry 2016

Supporting information for

Proton-coupled Electron Transfer in the Electrocatalysis of CO_2 Reduction: Prediction of Sequential *vs* Concerted Pathways using DFT

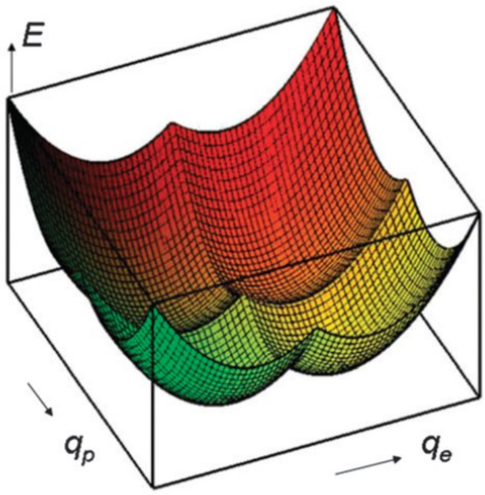

Figure S1. (reproduced from ref: Koper, M. T. M. *Phys. Chem. Chem. Phys.* **2013**, *15* (5), 1399) Topology of the potential energy surface (PES) along the PT and ET reaction coordinates (q_p and q_e) predicted by the theory with no solvent overlap (q_e and q_p orthogonal, $\lambda = 0$) and at the condition pH = pK_a (AH) and $E_{applied}$ = E_{CPET} = E_{ET}. The solvent overlap quantifies the coupling between the solvent modes along the PT and ET reaction coordinates. The SPET is kinetically favoured over CPET because the activation energy of the CPET ($(\lambda_e + \lambda_p)/4$, cf ref above) is always larger than for the individual steps ET ($\lambda_e/4$) and PT ($\lambda_p/4$).

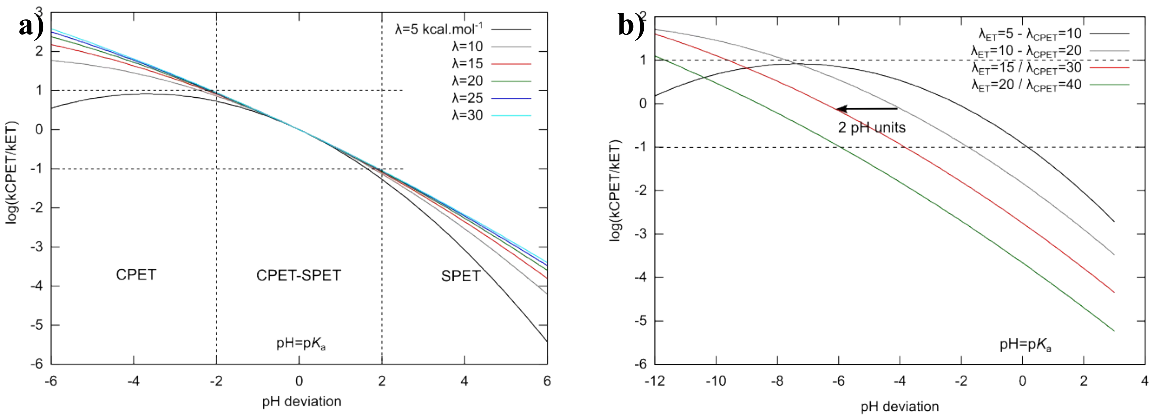

Figure S2. Quantitative analysis of the pH dependence of the competition between CPET and ET (SPET) steps. The analytical expressions corresponding to outersphere charge transfer are considered for the rate constant of the CPET and ET steps *(cf* eq. 1 and 3 in the manuscript) and the same preexponential factors are assumed for ET and CPET steps ($k°_{ET}=k°_{CPET}$). Different set of values for λ_{CPET} and λ_{ET} are studied. **a)** $\lambda_{ET}=\lambda_{CPET}$ corresponds to large favourable overlap between the reaction coordinates of ET and PT which leads to substantial negative values for the cross-reorganization energy $\bar{\lambda}$ (*cf* eq. 4 in manuscript). The region where both CPET and SPET pathways significantly take place (CPET-SPET) is defined for $\log(k_{CPET}/k_{ET})=\pm1$. For all λ values, this region is centred on the specific pH value pH=pK_a, for which $k_{CPET}=k_{ET}$ ($\log(k_{CPET}/k_{ET})=0$), and it approximately spans the pH range [pK_a-2:pK_a+2]. In the CPET-SPET region, the pH dependence is symmetric on both side of pH=pK_a. It is worth noting that for small λ values (λ=5 kcal.mol^{-1} on the plot), the inverted Marcus region may hindered the CPET pathway at relatively low pH. **b)** $2*\lambda_{ET}=\lambda_{CPET}$ is chosen to investigate the effect of the decrease of the overlap between ET and PT reaction coordinates ($\bar{\lambda}$ relatively small and negative or even positive if unfavourable overlap). The CPET-SPET region is substantially shifted to lower pH as the energy difference between λ_{CPET} and λ_{ET} increases ($\log(k_{CPET}/k_{ET})=0$ is shifted by ΔpH≈-2 for Δλ=+5 kcal.mol^{-1}). Furthermore, the pH range of this region tends to increase with Δλ. The trend observed for $2*\lambda_{ET}=\lambda_{CPET}$ are similar for λ_{ET}=cte and $\lambda_{CPET}=\lambda_{ET}+\Delta\lambda$ with Δλ=5, 10, 15, 20 etc.

a)

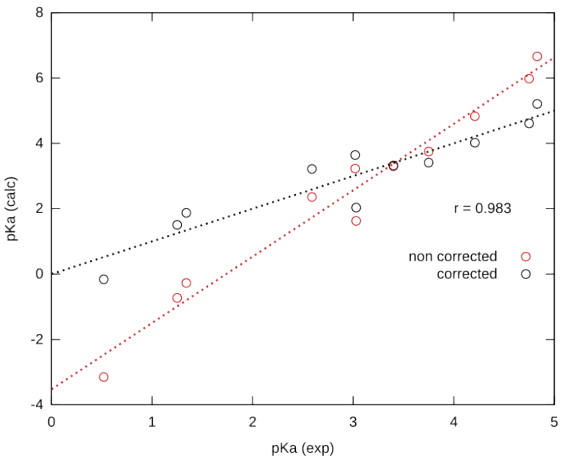

b)

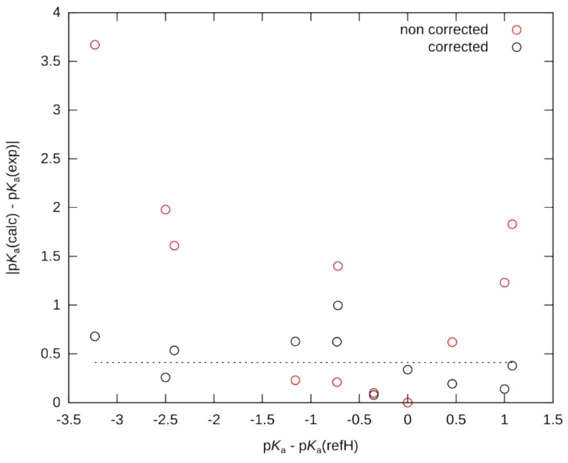

Figure S3. a) Plot of the predicted vs experimental pK_a values for a set of carboxylic acids before (red) and after correction (black) (carboxylic acids considered: trifluoroacetic, oxalic, difluoroacetic, fluoroacetic, fumaric, tartaric, D-malic, formic, succinic, butanoïc and acetic acids. Experimental pK_a values taken from the *Handbook of chemistry and physics 95th edition*). Before correction, the regression parameters are a=2.030 (slope) and b=-3.526 (offset). Corrected values are obtained with the addition of the term $corr(x) = (1 - a)x - b$ to each computed value. **b)** Plot of absolute errors of the predicted pK_a values against the pK_a difference with respect to the reference before correction (red) and after correction (black). Mean absolute error of the corrected values is displayed with a dashed line (~0.4 pK_a unit).

a)

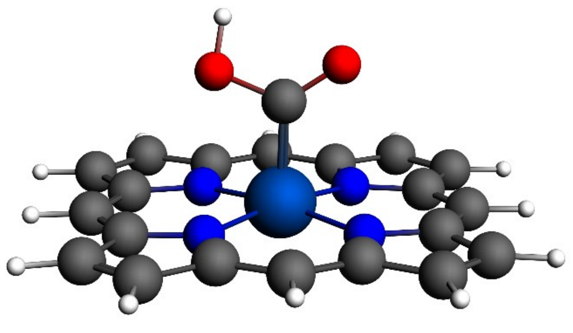

b)

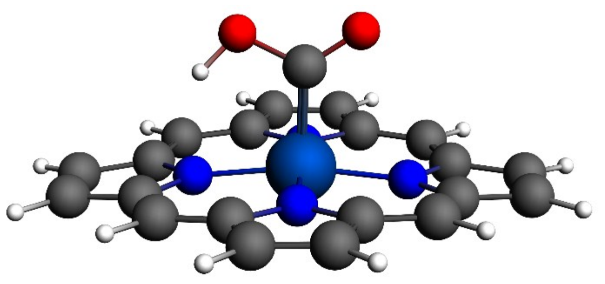

Figure S4. Structures of the two isomers of the neutral [CoP-COOH] intermediate **a)** without and **b)** with intramolecular hydrogen bond. Structures are very similar for the anionic [CoP-COOH] intermediate without explicit solvation.

a) **b)**

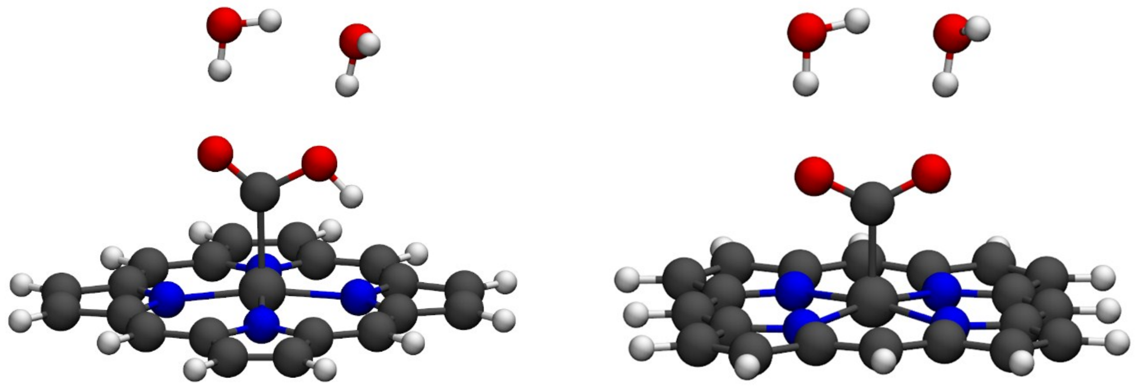

Figure S5. Structures of the anionic species **a)** [CoP-COOH] and **b)** $[CoP\text{-}CO_2]^-$ with two explicit water molecules.

	$[CoP\text{-}COOH]^-$	$[CoP\text{-}CO_2]^{2-}$	$[CoP\text{-}COOH]^-$ + 2 H_2O	$[CoP\text{-}CO_2]^{2-}$ + 2 H_2O
Co	0.39	0.34	0.39	0.35
P	-1.34	-1.55	-0.80	-1.51
COOH / CO_2	-0.05	-0.79	-0.52	-0.76

Table S1. Natural charges borne by the fragments, Cobalt (Co), Porphyrin (P), carboxyl (COOH) and CO_2 (CO_2) in $[CoP\text{-}COOH]^-$ and $[CoP\text{-}CO_2]^{2-}$ with and without explicit microsolvation.
